# The Role of the VEGF Family in Coronary Heart Disease

**DOI:** 10.3389/fcvm.2021.738325

**Published:** 2021-08-24

**Authors:** Yan Zhou, Xueping Zhu, Hanming Cui, Jingjing Shi, Guozhen Yuan, Shuai Shi, Yuanhui Hu

**Affiliations:** ^1^Guang'anmen Hospital, China Academy of Chinese Medical Sciences, Beijing, China; ^2^Beijing University of Chinese Medicine, Beijing, China

**Keywords:** VEGF, lipid metabolism, angiogenesis, lymphangiogenesis, inflammation, atherosclerosis, coronary heart disease

## Abstract

The vascular endothelial growth factor (VEGF) family, the regulator of blood and lymphatic vessels, is mostly investigated in the tumor and ophthalmic field. However, the functions it enjoys can also interfere with the development of atherosclerosis (AS) and further diseases like coronary heart disease (CHD). The source, regulating mechanisms including upregulation and downregulation, target cells/tissues, and known functions about VEGF-A, VEGF-B, VEGF-C, and VEGF-D are covered in the review. VEGF-A can regulate angiogenesis, vascular permeability, and inflammation by binding with VEGFR-1 and VEGFR-2. VEGF-B can regulate angiogenesis, redox, and apoptosis by binding with VEGFR-1. VEGF-C can regulate inflammation, lymphangiogenesis, angiogenesis, apoptosis, and fibrogenesis by binding with VEGFR-2 and VEGFR-3. VEGF-D can regulate lymphangiogenesis, angiogenesis, fibrogenesis, and apoptosis by binding with VEGFR-2 and VEGFR-3. These functions present great potential of applying the VEGF family for treating CHD. For instance, angiogenesis can compensate for hypoxia and ischemia by growing novel blood vessels. Lymphangiogenesis can degrade inflammation by providing exits for accumulated inflammatory cytokines. Anti-apoptosis can protect myocardium from impairment after myocardial infarction (MI). Fibrogenesis can promote myocardial fibrosis after MI to benefit cardiac recovery. In addition, all these factors have been confirmed to keep a link with lipid metabolism, the research about which is still in the early stage and exact mechanisms are relatively obscure. Because few reviews have been published about the summarized role of the VEGF family for treating CHD, the aim of this review article is to present an overview of the available evidence supporting it and give hints for further research.

## Introduction

Coronary heart disease (CHD) is the primary disease that threatens the safety of humans and burdens countries with heavy economic load. The morbidity of CHD has transcended 10% in some Western countries. The elderly over 65 years old are more susceptible to CHD, making the median incidence rate of the high-risk group 19.34% worldwide (1). The number of CHD patients in China has reached 11 million and the morbidity is still increasing (2).

The pathological basis of CHD is atherosclerosis (AS). AS is the asymmetric local thickening of the intima of the artery. Factors like lipid metabolism disorder, vascular endothelial cell (VEC) damage, inflammation, and immune dysfunction can promote the occurrence and development of AS, which then may lead to CHD (3). Lipid infiltration theory and inflammation theory are two classic theories about AS. The former claims that the elevated lipids such as low-density lipoprotein (LDL), very-low-density lipoprotein (VLDL), and its residues intrude into the artery wall and accumulate between smooth muscle cells (SMCs), collagen, and elastic fibers, which then cause proliferation of SMCs. Then, macrophages turn to foam cells by infiltrating lesions and devouring large amounts of lipids. AS plaques are formed eventually. Clinically, over 70% of CHD patients suffer from dyslipidemia concurrently (4). Active lipid-lowering therapy has been confirmed to reduce CHD risk and the target value of LDL-C is 1.8 mmol/L. It has been concluded that CHD risk will be reduced by 21% when LDL-C drops 1 mmol/L (5). While the inflammation theory suggests that AS is closely related to inflammatory response. Leukocyte recruitment and proinflammatory factors are mainly involved in the early steps of AS formation, including inflammation activating endothelial cells (ECs), monocytes invading lesions, and chemokines further promoting the recruitment of inflammatory cells to the endometrium, foam cells secreting inflammatory media, and macrophage apoptosis (6). Thus, inflammatory cells and immune cells form important parts of plaques, and the lift of inflammatory markers can be applied to assess the prognosis of patients with acute coronary syndrome (ACS) (7). Therefore, improving lipid metabolism and alleviating inflammatory response play a crucial role to prevent and control CHD.

**Figure 1:**
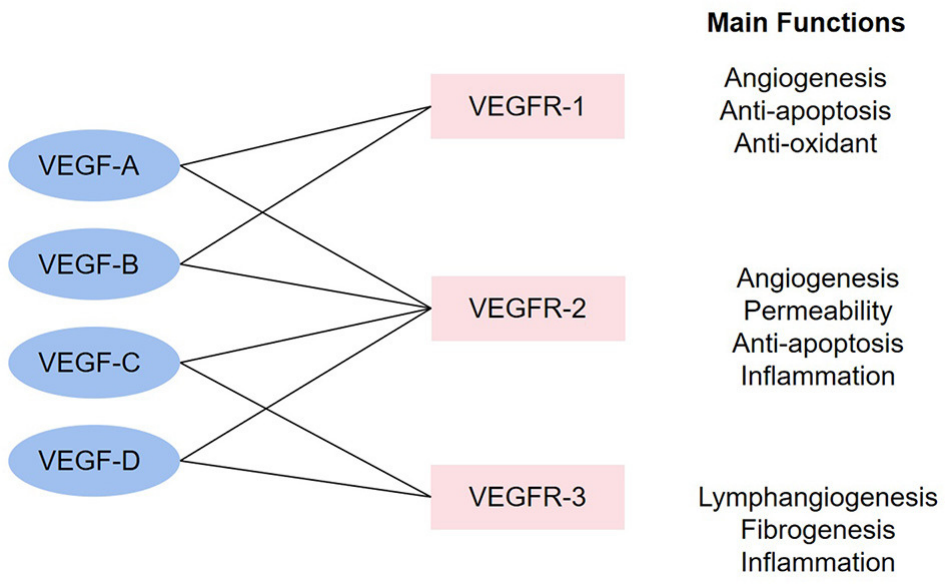
The combinations in the VEGF family and main functions. The linkage between VEGF factors and VEGFR represents that the factors can combine with the receptors. The main functions stand for the functions after the activation of each receptor.

The vascular endothelial growth factor (VEGF) family governs functions including promoting angiogenesis, promoting lymphopoiesis, regulating inflammation, resisting oxidative stress, and regulating lipid metabolism, which presents potential therapeutic value for CHD. The VEGF family consists of the following members: VEGF-A, VEGF-B, VEGF-C, VEGF-D, VEGF-E (virus source), VEGF-F (snake venom source), placenta growth factor (PlGF), and EG-VEGF. VEGF receptors (VEGFRs) include VEGFR-1, VEGFR-2, and VEGFR-3. VEGFR-1 and VEGFR-2 are mainly expressed on VECs, while VEGFR-3 is mostly expressed on lymphatic endothelial cells (LECs) (8). These factors perform functions *via* combining to related receptors (see Figure 1). Recently, a large number of studies have shown the capacity of the VEGF family to interfere with lipid metabolism, especially through the regulation of lymphatic system. This system is a one-way transport pathway from extracellular space to venous system, which participates in the absorption of lipid in the gastrointestinal tract. In particular, VEGF-C and VEGF-D, as significant regulatory factors of lymphatic system, influence lipid level from this aspect. As for inflammation, dilation and proliferation of lymphatic vessels mediated by VEGF family provide an exit for local accumulated inflammatory media, thus inhibiting inflammatory response and AS progress. The VEGF family can also prevent AS by promoting angiogenesis and regulating oxidative stress. In terms of angiogenesis, coronary artery AS can lead to ischemia and hypoxia in local myocardium. The expression of some factors in the VEGF family would be enhanced in such environment, thus promoting the proliferation and migration of ECs and forming compensatory neovascularization. Oxidative stress has been proved to be an accelerator for AS, and the balance effect of the VEGF family on oxidation–reduction may also slow down the progress of AS.

In conclusion, the multiple perspectives and targets presented by the VEGF family predict their ability to regulate the progress of AS and provide potential therapeutic strategies for treating cardiovascular diseases. Until now, no relevant review about the biological effects of the VEGF family on CHD has been published. Therefore, based on previous studies, we summarized the known mechanisms of the VEGF family on blood vessels and lymphatic vessels, as well as their influence on inflammation, apoptosis, fibrogenesis, and lipid metabolism. The study incorporates and arranges information from four main aspects, the origin of the VEGF family, up/downregulation mechanism, receptors/target cells, and functions, aiming to provide a literature review for further study to explore more effective applications of the VEGF family to CHD.

## VEGF-A

VEGF-A, namely, VEGF, is one of the polypeptide proteins and the most established factor in the VEGF family. By combining with VEGFR-1 and VEGFR-2, it mediates angiogenesis, vascular permeability, and inflammation, making itself known as a vascular permeability factor (VPF) (9). Physiological angiogenesis, that is, forming blood vessels in the process of tissue revascularization, involves the cascade of multiple signals. VEGF-A, the core of the process, is crucial for angiogenesis and the functions of ECs, while pathological angiogenesis is a marker of inflammatory and ischemic diseases. A clinical study has shown that the level of VEGF-A is independently associated with microvascular occlusion (MVO) during ST segment elevation myocardial infarction (STEMI), and correlated with mid-term left ventricular ejection fraction (LVEF). Therefore, VEGF-A can be regarded as a biomarker of MVO in STEMI patients and can be applied for prognosis stratification (10). However, the role of VEGF-A to AS is controversial; for instance, the increase of VEGF-A can both promote vascular proliferation to compensate for myocardial hypoxia and enhance vascular permeability to speed up inflammation.

**Figure 2:**
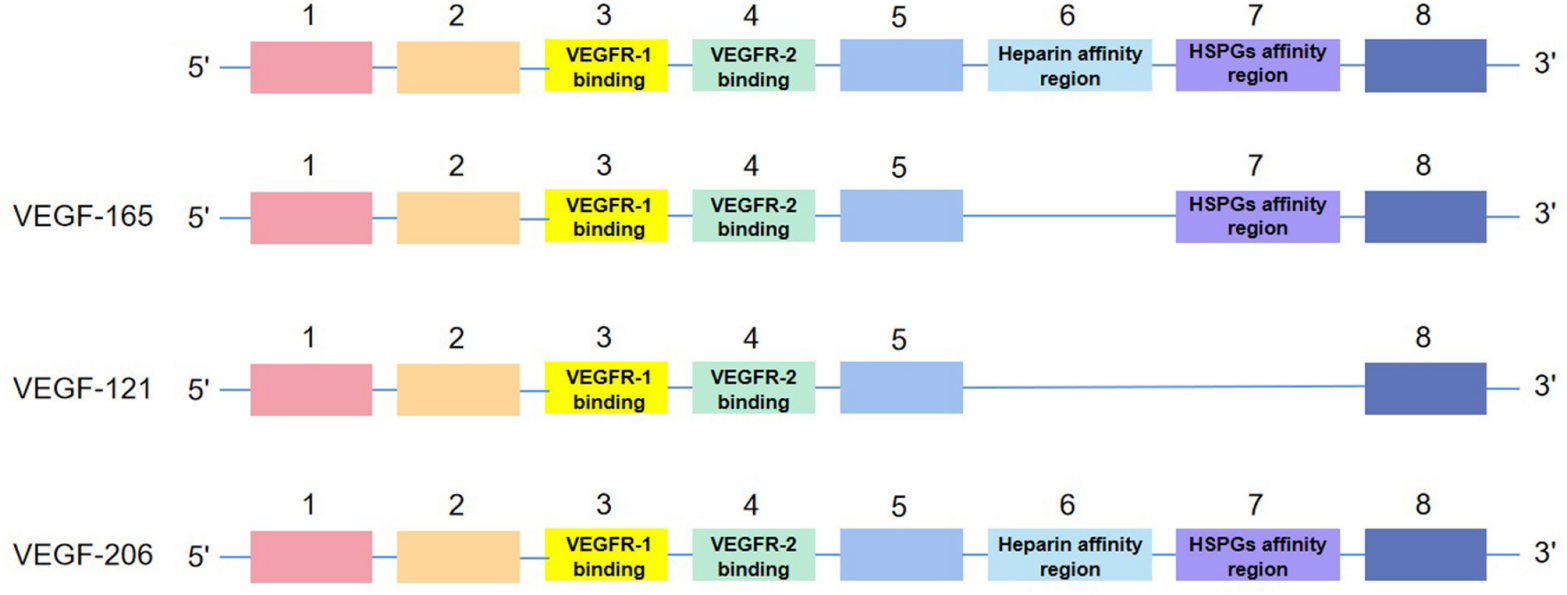
The structure of isoforms of VEGF-A. VEGF-165, VEGF-121, and VEGF-206 are examples of the structure.

Human VEGF-A, composed of eight exons separated by seven introns, presents subtypes with different length, such as VEGF165, VEGF121, and VEGF206, after the splicing of alternative VEGF mRNA. All subtypes contain the same regions, exons 1–5 and 8. Their differences in exons 6–7, namely, heparin affinity and heparin sulfate proteoglycan (HSPG) affinity regions, contribute to their different biological characteristics (11) (see Figure 2). Meanwhile, as a double-edged factor, it comprises isoforms that antagonize with each other, which accentuates its arcane mechanisms. Concretely, anti-angiogenic VEGFxxxb isoforms oppose to VEGFxxxa isoforms and reduce VEGFR-2 activation (12). Like VEGF165a, the most active subtype in vascular reconstruction is inhibited by VEGF165b (13).

### Source of VEGF-A

**Figure 3:**
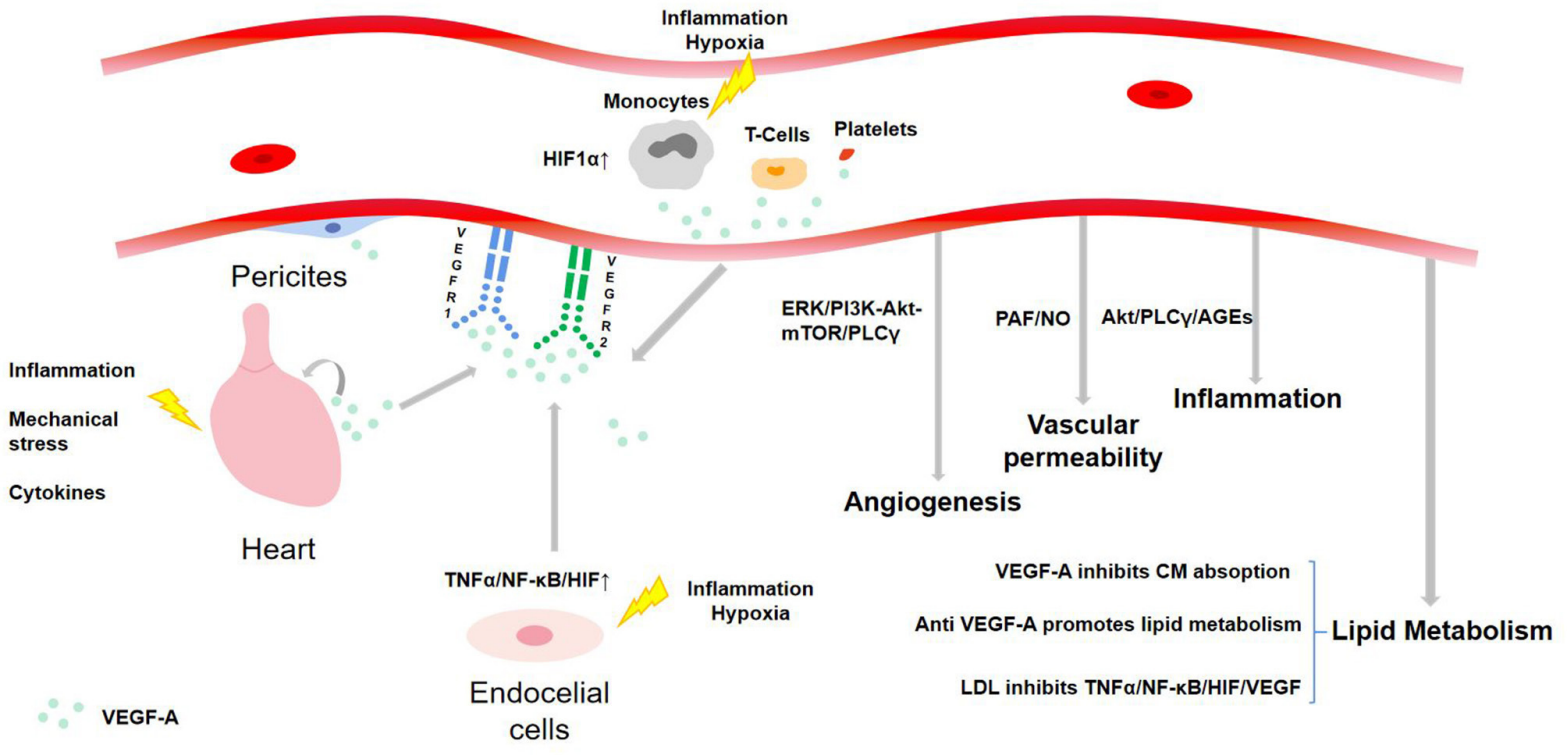
The source and regulation mechanism of VEGF-A with functions.

VEGF-A is expressed in most systems in the human body. In the cardiovascular system, pericytes, ECs, and angioblasts are the primary expression sites. Under hypoxia and inflammatory environment, VEGF-A can also be produced by blood cells including monocytes, activated T cells, neutrophils, dendritic cells, and platelets (8). In CHD patients, VEGF-A will be secreted by myocardium due to local inflammation, mechanical stress, and cytokines, resulting in myocardial deformation, contraction, and impaired recovery (14) (see Figure 3). Other cells, such as terminal chondrocytes, hypertrophic chondrocytes, tumor cells, keratinocytes, and retinal pigment epithelial cells, can also produce VEGF-A under certain circumstances.

### Mechanisms Regulating the Level of VEGF-A

#### Upregulation Mechanism of VEGF-A

It is widely held that in cardiovascular diseases, cardiomyocytes and ECs are often exposed to hypoxia and inflammation, which stimulate hypoxia-inducible factors (HIFs). HIFs can upregulate various pro-angiogenic factors *via* the HIF-1α pathway, especially VEGF to promote angiogenesis. Increased VEGF-A favors the proliferation of VECs, improves vascular permeability, and restores the integrity of endothelium and vascular function. Therefore, it compensates for ischemia and hypoxia, and protects the injured myocardium (15). In addition to the typical pathway, many recent studies have uncovered other mechanisms of upregulating VEGF-A. For example, a study published on *Nature* claimed that lack of oxygen and nutrition caused the activation of transcription coactivator PGC-1, a major regulator of mitochondrial function, and promoted angiogenesis by lifting the expression of VEGF. PGC-1 further synergistically activated orphan nuclear receptor ERR-α to control an angiogenesis pathway independent of HIFs (16). Jin found that hypoxia also induced ECs to produce TNFα, which suggested that the autocrine cycle could activate HIFs by a NF-κB dependent process, to upregulate VEGF and promote angiogenesis. This forms the TNFα/NF-κB/HIF/VEGF signaling cascade in ECs (17). Longchamp discovered that thioamino acid restriction was a trigger of angiogenesis as it enhanced the expression of VEGF and capillary density of skeletal muscle in mice through GCN2/ATF4 amino acid starvation pathway independent of hypoxia or HIF-1α. In addition, hydrogen sulfide (H2S) was observed to promote angiogenesis by inhibiting mitochondrial electron transport and oxidative phosphorylation, resulting in increased glucose uptake and ATP production in glycolysis (18). Guo found that in AS plaque, CD163+ macrophages contributed to the expression of VEGF-A through the CD163/HIF1α/VEGF-A pathway (19). Annexin A1 (AnxA1) directly led cardiac macrophages to polarize to angiogenic and repair phenotypes, and stimulated cardiac macrophages to release large amounts of VEGF-A, thus inducing angiogenesis and cardiac repair (20). By contrast, one study revealed that mild hypoxia (14% O_2_) did not induce coronary angiogenesis or VEGF-A expression in hearts of developing mice (21).

In addition to environmental factors, some drugs have also been confirmed to protect myocardium by upregulating VEGF-A. For instance, hydroxysafflor yellow A in safflower improved the function of endothelial progenitor cells (EPCs) and increased VEGF-A in ECs in MI model mice *via* the HO-1/VEGF-A/SDF-1α signaling cascade, which then significantly recovered cardiac hemodynamics induced by ischemia, improved survival rate, and alleviated myocardial injury (22, 23). *Pueraria* and *Salvia miltiorrhiza* extracts promoted angiogenesis by upregulating the VEGF/VEGFR2 pathway, thus preserving myocardium of MI model rats (24). Salidroside increased the expression of HIF-1α and then the level of VEGF to inhibit necrosis and apoptosis of cardiomyocytes induced by hypoxia (25).

#### Downregulation Mechanism of VEGF-A

The above TNFα/NF-κB/HIF/VEGF signaling cascade in ECs has been proved to be destroyed by LDL, because it can suppress TNFR1, prevent the expression of HIFs in ECs exposed to hypoxia or TNFα, and thus decrease VEGF. Similarly, knocking out HIF-1α or HIF-2α also significantly reduced the production of VEGF induced by hypoxia, thereby inhibiting angiogenesis (17). In addition, E2F1 transcription factor inhibited the upregulation of VEGF and PIGF through p53-dependent and -independent mechanisms, respectively, thus limiting cardiac neovascularization and functional recovery after MI (26). In the rat model of myocardial reperfusion injury, overexpression of ANXA1 inhibited inflammatory factors and the infiltration and apoptosis of polymorphonuclear neutrophils (PMNs), which further downregulated STAT3 signaling pathway to hamper the expression of VEGF (27). Many studies unearthed that lipid-lowering drugs could also downregulate VEGF. For example, high-dose rosuvastatin monotherapy lowered VEGF in patients with dyslipidemia, while low-dose rosuvastatin and polyunsaturated fatty acids significantly increase VEGF (28). After undergoing percutaneous coronary intervention (PCI), VEGF level climbed up at first in MI patients, which could reflect transient ischemia and lead to neovascularization in AS plaque, while later rosuvastatin treatment reduced the previously elevated VEGF (29). Simvastatin combined with coniferin were claimed to inhibit the expression of VEGF and endothelin (ET) and increase the level of NO and the activity of superoxide dismutase (SOD) in ApoE –/– mice fed with a high-fat diet (HFD). The levels of serum total cholesterol, triglyceride, and LDL-C were significantly reduced, thus restraining the development of AS (30).

### Receptors and Target Cells

#### VEGFR-1

VEGFR-1 is a member of receptor tyrosine kinase family (RTKs), with high affinity with VEGF-A but 10 times weaker kinase activity compared to VEGFR-2. It is mainly expressed in ECs and also in other cells including inflammatory cells, monocytes/macrophages, vascular smooth muscle cells (VSMCs), bone marrow-derived hematopoietic progenitor cells, and cardiac fibroblasts in myocardial infarction (MI) (31). VEGFR-1 regulates the migration of these cells and plays an important part in angiogenesis. VEGFR-1 contains three domains, intracellular domain, extracellular domain, and transmembrane domain, the latter two relating to angiogenesis (32). VEGFR-1 competes with VEGFR-2 for VEGF-A depending on high affinity, but due to its low kinase activity, it can inhibit angiogenesis in some condition. In the embryonic stage, VEGFR-1 restrains angiogenesis by “seizing” VEGF-A, while in adulthood, it ameliorates macrophage function to adjust the development of inflammatory diseases, cancer metastasis, and AS in a creatine kinase-dependent manner. The activity of VEGFR-1 tyrosine kinase affects pathological angiogenesis and inflammation. Mice with VEGFR-1 which lacked tyrosine kinase region showed milder inflammatory response (33).

#### VEGFR-2

VEGFR-2, another member of RTKs, is mainly expressed in ECs of blood vessels and lymphatic vessels, and weakly spotted in neurons, tumor cells, and hematopoietic cells. The combination of VEGF-A and VEGFR-2 is key to controlling angiogenesis, vascular permeability, and inflammation. VEGFR-2, like VEGFR-1, contains three domains. When VEGF-A binds to the extracellular domain of VEGFR-2, it can lead to the autophosphorylation of tyrosine residues and energizing signaling pathways that can promote the proliferation of ECs. Unlike other RTK family members that regulate the Ras pathway, VEGFR-2 mainly activates MAPK and DNA synthesis using the phospholipase-Cγ-protein kinase-C pathway (34).

### Functions of VEGF-A

#### Angiogenesis

VEGF-A-induced pathways are strictly regulated in space and dynamics to coordinate the proliferation, migration, and invasion of ECs into surrounding tissues, leading to the formation of lumen structures and further neovascularization. The binding of VEGF to VEGFR-2 causes receptor endocytosis and activates a variety of downstream signaling pathways, including ERK, PI3K-Akt-mTOR, and PLCγ, which promotes the proliferation and migration of VECs and vascular remodeling. The proliferation of ECs is primarily stimulated by ERK and PI3K/Akt signaling pathways. Furthermore, the activation of ERK is mainly influenced by upstream signals including PLCγ, IQGAP1, FAK, and Gab1, while PI3K is affected by Shb, IQGAP1, AXL, Gab1, and FAK (9). The migration of ECs is mainly regulated by G protein in Rho family stimulated by PI3K. The invasion of ECs occurs due to the release of matrix metalloproteinases (MMPs), which degrade the basement membrane and extracellular matrix to allow the migration of new ECs, leading to the formation of capillary sprouts. VEGF-A has been proved to induce MMP, MMP-2, MMP-9, and urokinase plasminogen, and the potential mechanism may be the activation of β-Kartin and NF-κb (35).

It has been found that multiple proteins that work on the above pathways can affect angiogenesis of VEGF-A. For example, N-myc downstream regulatory gene 1 (NDRG1) formed a complex with PLCγ1 through its phosphorylation site. PLCγ1 inhibited angiogenesis induced by VEGF-A, which indicated that NDRG1 plays an important role in VEGF-A-induced angiogenesis through PLCγ1 signal transduction (36). In breast cancer patients, downregulation of angiotensin-converting enzyme 2 (ACE2) checked the expression of VEGF-A and deactivated the phosphorylation of VEGFR2, MEK1/2, and ERK1/2 in human umbilical vessel endothelial cells (HUVECs), thereby inhibiting angiogenesis through the VEGF-A/VEGFR-2/ERK pathway (37). The activation of mineralocorticoid receptor (MR) inhibited VEGF-induced gene expression, resulting in the disorder of angiogenesis-related gene network in ECs, which destroyed the ability of VEGF to induce angiogenesis (38). Increased vascular permeability also promoted vascular growth by promoting the rapid growth of extracellular proteases required for angiogenesis. In addition, leukocyte extravasation helped early angiogenesis by enhancing the local production of cytokines/chemokines and release of proteases (9). However, a recent study has shown that VEGF-A also induced vascular degeneration, which was regulated by phosphatidylinositol metabolic cycle controlled by CDP diacylglycerol synthase-2 (CDS2). ECs with mutant CDS2 suppressed vascular degeneration through reverse migration and apoptosis after being stimulated by VEGF-A (39).

At present, studies about VEGF (VEGF-A) and CHD mainly focus on angiogenesis of VEGF. Zhao found that the extracts of Radix Puerariae and Radix Salviae Miltiorrhizae could induce salient angiogenesis in HUVECs by regulating the VEGF/VEGFR-2 signaling pathway. Meanwhile, the infarct size of the treatment group was smaller than that of the model group, with significantly increased expression of related proteins. It was then suggested that the extracts may protect myocardial cells after MI by promoting angiogenesis *via* the VEGF/VEGFR-2 pathway (24). Li found that Shexiang Xintongning could promote angiogenesis by activating VEGF/VEGFR2 and ERK1/2 pathways to protect HUVECs from oxidative stress, reduce infarct size, and prevent AS plaque rupture-induced worsening CHD (40). Xu found that Shenzhu Guanxin granules could upregulate the expression of PECAM-1/CD31 and VEGF in a dose-dependent manner to improve cardiac hemodynamic function and reduce MI area (41). Zhang claimed that Shexiang Baoxin Pill could significantly enhance the expression of VEGF so as to promote angiogenesis, improve microvascular count, and reduce the size of MI (42). Besides, hampering the activity of VEGF-A and angiogenesis can also work in clinical practices. For example, cryptotanshinone, the active component of *S. miltiorrhiza*, has been found to check VEGF-induced VEGFR2 phosphorylation and its downstream Src/FAK and ERK1/2 signaling pathways, including p-ERK1/2, p-p90RSK, py416-Src, and pY576/577-FAK proteins, which are responsible for EC migration, proliferation, and survival. This component effectively inhibited VEGF-induced angiogenesis, highlighting the therapeutic potential for treating angiogenesis-related diseases (43). The development of abdominal aortic aneurysm (AAA) is characterized by VEGF-induced angiogenesis through cyclooxygenase-2 (COX-2), while quercetin can inhibit COX-2 and reduce HIF-1α/VEGF signaling-related angiogenesis, thereby inhibiting aneurysm growth (44). A kind of marine fungus extract, asperchalasine A, can inhibit angiogenesis by downregulating VEGF, p-p38, ERK, p-VEGFR-2, and p-Akt signaling pathways (45). Mesenchymal stem cell processed by atorvastatin can activate the Akt/eNOS pathway, increase VEGF level, and promote vascular proliferation (46). In addition to pharmacological studies, the level of VEGF in CHD patients can be significantly raised after physiological ischemia training, indicating that such training in an appropriate period may ameliorate blood pressure and cardiac structure by increasing the secretion of VEGF (47).

#### Vascular Permeability

High vascular permeability caused by twisted EC cell–cell junction is associated with no reflow phenomenon after reopening occluding vessels in CHD patients, which also accounts for the main cause of death in these patients (48). Angiogenesis and vascular permeability are essential for normal tissue homeostasis. Chronic high permeability can lead to destructive tissue edema, like edema in CHD patients due to permeability imbalance. The VEGF-A/VEGFR-2 pathway is that by which VEGF-A regulates vascular permeability. The synthesis of endogenous endothelial platelet activating factor (PAF) and nitric oxide (NO) contributes to vascular permeability mediated by VEGF-165 (49). Inhibiting vascular permeability regulated by VEGFA/VEGFR2 was found to reduce edema in MI model mice without affecting the vascular density associated with survival rate after MI (50).

The two mechanisms of vascular permeability are the formation of transcellular pores and transient opening of paracellular junction. Firstly, transcellular pores such as vesicle-vacuole organelles (VVOs) are assembled by lipid microstructural domains and cross the vein endothelium. VEGFR-2 is located in the cave membrane and forms a complex with caveolin-1 (Cav-1) through its C-terminal tail. Secondly, permeability induced by VEGF-A depends on the transient opening of adhesion junction and tight junction. Vascular endothelial cadherin (VE-cadherin) is a kind of adhesion protein that controls vascular permeability. Adhesion is a cell–cell interaction, mainly created by the intermolecular binding of VE-cadherin. VEGF-A causes the short opening of endothelial cell–cell connections and dissolve the adhesion junction and tight junction. VEGF-A can dissolve the VE-cadherin complex by activating Src and Yes protein, which both then phosphorylate VE-cadherin and β-Kartin to improve vascular permeability. The third mechanism for the dissociation of VE-cadherin from the junction involves vessel endothelium-phosphotyrosine phosphatase (VE-PTP), which has been confirmed to be related to VEGFR-2 and VE-cadherin. The exact mechanism has not been elucidated, and one possibility is that the interaction between VE-PTP and angiopoietin receptor Tie2 affects angiopoietin 1 and 2. The decrease of tyrosine phosphatase activity may be conducive to the increase of tyrosine phosphorylation of VE-cadherin, thus accelerating the opening of the connection. Shb deficiency may cause abnormal signal conduction of G protein in Rho family and then reduce vascular permeability induced by VEGF-A and dissociate VE-cadherin at the adhesion junction. Vascular permeability induced by VEGF165 requires VEGFR-2 and NRP1, including NRP1 cytoplasmic domain (NCD). In the VEGF165 binding receptor complex, NCD promotes the activation of ABL kinase, while ABL kinase then activates the SRC family kinase (SFK) recruited by VEGFR-2. In the mice model of choroidal neovascularization, the loss of NCD reduced the leakage of blood vessels without affecting neovascularization. The result demonstrated that the targeted regulation of NRP1 or NCD could reduce edema induced by VEGF165 without changing the growth of blood vessels, which was made a potential strategy for the treatment of neovascular diseases (51). Akt, the downstream protein of the VEGF signaling pathway, can activate eNOS and produce NO to expand blood vessels and enhance vascular permeability. The active Akt can also promote vascular permeability independently from the VEGF pathway, which involves the activation of mTOR and phosphorylation of eNOS. The VEGF-dependent activation of eNOS can be achieved by the direct binding of eNOS with Ca2+/calmodulin (CAM) or by phosphorylation of Akt and AMP-activated protein kinase, CaM-dependent kinase II, and PKA (52). In addition, VEGFR-3 can inhibit the expression of VEGFR-2 and the activity of VEGF/VEGFR2 pathway in VECs, so as to prevent excessive vascular permeability. It was found that long-term exposure to VEGF-A inhibited the release of Ca2+ mediated by agonists and the further activation of the IKCa channel. The depressed channel promoted vasodilation through endothelial-dependent hyperpolarization (EDH) of the resistance artery in mice. VEGF-A downstream of MEK signaling weakened the above vasodilation response of ECs, indicating that VEGF-A played a new role in the resistance artery and provided a novel aspect of treating cardiovascular disease with VEGF-A (53).

In drug research, melatonin was observed to inhibit VEGF-induced monolayer permeability of HUVECs, which was related to the phosphorylation of VE-cadherin. Blocking PI3K/AKT and MEK/ERK pathways could inhibit the permeability of HUVECs, implying that AKT (Ser473) phosphorylation might be a key event in the change of monolayer permeability (54). Rosiglitazone activated PI3K-AKT or protein kinase C (PKC) β to promote the migration of ECs and induce vascular permeability. In addition, rosiglitazone might promote angiogenesis and vascular leakage by increasing the expression of VEGF and suppressing the expression of tight junction proteins (JAM-A and ZO-1), while inhibiting AKT reached the opposite (55). Excessive vascular permeability occurs during inflammation. Anti-VE-cadherin monoclonal antibodies (mAbs) activated cell adhesion and inhibited the increase of monolayer permeability of ECs induced by thrombin receptor activating peptide-6 (TRAP-6). Among mAbs, 8A12c and 3A5a could decrease vascular permeability, while the inhibitory mAb 2E11d could enhance vascular permeability. Activated mAbs could also depress vascular permeability induced by VEGF or TNF-α (56).

#### Inflammation

Inflammation, often accompanied by neovascularization, is a crucial feature of inducing pathological angiogenesis. Therefore, higher vascular permeability is often viewed together with the recruitment of inflammatory cells. VEGF can promote the recruitment of inflammatory cells, which are mostly composed of neutrophils and macrophages and, to a lesser extent, of T cells and B cells (57). In addition, VEGF-A-induced PLCγ activation can increase Ca^2+^, which will activate the transcription factor NFAT through calcineurin. This way can not only energize angiogenesis but also induce inflammation gene expression pattern similar to IL-1β. Activating NF-κB downstream of AKT can also cause inflammatory type response, leading to changes in gene expression profile. Advanced glycation end products (AGEs) can activate RAGE-NF-κB pathway in human synovial cells to induce the expression of cyclooxygenase-2 (COX-2) and VEGF, and then promote the production of prostaglandin-E2 (PGE2), VEGF, IL-6, and MMP-13 to exacerbate inflammation (58). Therefore, depressing VEGF may play an anti-inflammatory role and the value of VEGF can reflect the level of local inflammatory response (59). When treated by lipopolysaccharide (LPS), a kind of inflammatory mimic, human microvascular endothelial cells (HMEC-1) were found to secrete VEGF and prostaglandin (bFGF). However, further use of ibuprofen only reduced bFGF, but further enhanced the level of VEGF (60). In the MI mouse model, resveratrol (RSV)-loaded scaffolds increased the expression of cTnT, Cx-43, Trx-1, and VEGF; decreased inflammatory cell infiltration; and improved vascular network formation after MI (61). Therefore, higher VEGF level does not ensure increased inflammatory response and worse prognosis because complex regulatory mechanisms will form a comprehensive impact.

### The Relation Between VEGF-A and Lipid Metabolism

VEGF-A has been shown to induce the accumulation of triglycerides in large lipoprotein granules, including VLDL, chylomicrons (CM), and residues. A VEGF-A transfer study found that VEGF-A decreased the activity of plasma lipoprotein lipase (LPL), and each model group showed the change of promoting AS in lipid spectrum (62). VEGF-A can also regulate lipid metabolism in intestinal lymphatic vessels through the VEGF-A/Nrp1/VEGFR-1 pathway (63). The mechanism lies in that the deletion of Nrp1 and VEGFR1 can inhibit CM entering chylous duct, promote the combination of VEGF-A and VEGFR-2, enhance the biological activity of VEGF-A, and then inhibit CM absorption (64). Anti-VEGF therapy can also affect serum lipids. The most common clinical treatment related to VEGF is antiangiogenic therapy applied in human tumors. In addition to increasing glycolysis and lactate production, bevacizumab, an anti-VEGF neutralizing antibody, can upregulate lipid metabolism pathways, resulting in spectacular changes in tumor lipid mass spectrometry, including increased triglyceride levels and decreased lipid chain saturation (65).

At the same time, the value of blood lipid can also regulate the level of VEGF-A and affect its biological activity. HFD has been proved to increase serum VEGF level (66). There is a self-regulating TNFα/NF-κB/HIF/VEGF signaling network in ECs that can mediate and regulate angiogenesis in a hypoxic environment, while elevated LDL can hamper angiogenesis by destroying this network, which may lead to decompensation of CHD patients (17). Adipocyte-conditioned medium from human adipocytes enhanced the expression of VEGFR-1 and VEGFR-2, and the secretion of VEGF by VSMCs. The increased VEGF then stimulated the proliferation of VSMCs. Therefore, VEGF may associate the inflammation of adipose tissue with the increased proliferation of VSMCs (67).

## VEGF-B

VEGF-B discovered in 1995 is a homolog of VEGF, with which it shares high sequence homology, but it can only function by binding with VEGFR-1. There are two subtypes of VEGF-B, VEGF-B167 and VEGF-B186. As the main subtype, VEGF-B167, accounting for over 80% of the total number of VEGF-B, is expressed in most tissues. It can bind to cell surface or extracellular matrix (ECM) elements and easily interact with neuropeptide-1 (NP-1). VEGF-B186 exists in free state and will interact with NP-1 only when it undergoes proteolysis. VEGF-B conduces to the development of cardiovascular system and the formation of embryonic myocardium, but pertinent research about the effect and mechanism of VEGF-B is more limited than that of VEGF-A (68, 69).

### Source of VEGF-B

VEGF-B is mainly expressed in myocardium, coronary artery smooth muscle cells, ECs, and pancreas, as well as in lung, kidney, gallbladder, and fat (70, 71).

### Mechanisms Regulating the Level of VEGF-B

#### Upregulation Mechanism of VEGF-B

Vagal neurotransmitter acetylcholine upregulated the expression of VEGF-A and VEGF-B through the m/nACh-R/PI3K/Akt/Sp1 pathway in ECs. VEGF-B induced VSMC proliferation and EC migration, and finally promoted angiogenesis/arteriogenesis to repair MI heart under the synergistic effect with VEGF-A (70). RSV pretreatment increased the expression of VEGF-B, p-eNOS, and p-AMPK and the production of NO in rats treated with subcutaneous injection of isoproterenol (ISO) (72).

#### Downregulation Mechanism of VEGF-B

Some antitumor drugs can introduce cardiotoxicity by downregulating the expression of VEGF-B in cardiomyocytes. For example, doxorubicin (DOX) is a broad antineoplastic drug, which can suppress the expression of VEGF-B, while RSV significantly attenuated DOX-induced cardiotoxicity by lifting VEGF-B (73).

### Receptors and Target Cells

VEGF-B functions on assorted kinds of cells and tissues in humans by binding to VEGFR-1. Not only can it promote the survival of VECs, but it also contributes to the survival of pericytes, smooth muscle cells (SMCs), and vascular stem cells/progenitor cells. *In vivo*, VEGF-B has been proved to inhibit choroidal and retinal neovascularization by regulating the expression of multiple vascular pro-survival genes (74).

### Functions of VEGF-B

#### Angiogenesis

VEGF-B bears a weak angiogenesis capacity, but still plays a significant role in vascular growth and survival. VEGF-B is saliently expressed in tissues with high metabolic activity like heart. Studies have shown that overexpression of VEGF-B in myocardium could promote neovascularization directly from the ventricle, maintain the connection with coronary vessels in subendocardial myocardium, accelerate the proliferation of subendocardial myocardial endothelium, and rescue the structure and function of myocardial tissue after MI (75). VEGF-B in cardiomyocytes can increase VEGF signal through VEGFR-2, activate Erk1/2, and promote vascular growth. VEGF-B can upregulate Akt and mTORC1 pathway, downregulate AMPK pathway, and reorganize the metabolic process of myocardium, which is conducive to glucose oxidation and macromolecular biosynthesis. Therefore, VEGF-B can be applied to increase the coronary vascular system in patients with ischemic heart disease and reprogram myocardial metabolism to improve cardiac function. However, the expression of VEGF-B decreases in CHD patients (76). A number of studies have revealed that adenovirus (AD) VEGF-B186 gene transfer caused significant angiogenesis in myocardium, increasing myocardial perfusion without increasing coronary steal effect. Some mechanisms may be related to G protein signaling. AdVEGF-B186 also enjoys tissue specificity and high efficiency on ischemic myocardium. It can also inhibit myocardial apoptosis and regulate myocardial metabolism, becoming a potential drug for the treatment of myocardial ischemia (77, 78).

#### Antioxidation

Oxidative stress can cause damage to VECs and increase the risk of CHD. It has been found that VEGF-B can lift many key antioxidant genes by binding with VEGFR-1, including Gpx1, one of the downstream effectors, in order to exert antioxidant effect (79). RSV is a natural antitoxin in polyphenol plant. Pretreatment with RSV significantly reduced the production of superoxide and malondialdehyde (MDA) in ISO-treated rats and increased superoxide dismutase (SOD) to play an antioxidant role. Besides, RSV prevented the adverse changes of infarct size and apoptosis in ISO-treated rats. Therefore, RSV may protect myocardium after MI through the VEGF-B/AMPK/eNOS/NO signaling pathway (72).

#### Anti-apoptosis

VEGF-B has a strong anti-apoptotic effect on both myocardial cells cultured *in vitro* and myocardial cells after MI. It has been found that VEGF-B could induce specific gene expression profiles of compensatory hypertrophy by activating VEGFR-1. Concretely, VEGF-B activated αMHC; inhibited βMHC and bone αactin; increased SERCA2a, RYR, PGC1α, and cardiac natriuretic peptide transcripts; and ultimately prevented the loss of myocardial mass and maintained myocardial contractility (80).

### The Relation Between VEGF-B and Lipid Metabolism

VEGF-B enjoys effective lipid-lowering function. By binding with VEGFR-1 and NP-1, VEGF-B transcriptionally regulates vascular fatty acid transporters and specifically controls the uptake of fatty acids by ECs, which promotes the uptake and transcytosis of fatty acids from blood flow to lower tissues including heart and skeletal muscle, so as to reduce the circulating lipid. Moreover, the lipid uptake of ECs is closely related to the lipid utilization of mitochondria (81). The lipid regulation function of VEGF-B was elucidated by a research in which ceramide enhanced and triglyceride decreased in the heart of VEGF-B transgenic mice (82). VEGF-B also induced the expression of carnitine palmitoyltransferase-1 (CPT-1), and phosphorylated ACC and AMPK in the muscle and liver of mice fed with HFD to lower lipid (83). For type 2 diabetes mellitus (T2DM) patients, increased lipid handling and lipid toxicity may lead to dysfunction of islet β cells. Systemic inhibition of VEGF-B signal in T2DM patients prevented lipid accumulation, improved insulin sensitivity and glucose tolerance, and reduced the content of islet triglyceride. Mice, which owned islets β cells selectively had VEGF-B cleared, expressed higher islet genes. However, glucose homeostasis and islet lipid uptake were not affected by the clearance of VEGF-B (84). Another study also showed that inhibition of VEGF-B signal reconstructed membrane cholesterol level and restored glucose uptake, while enhancement of VEGF-B signal weakened LDL receptor (LDLR)-mediated cholesterol uptake, resulting in lower plasma membrane cholesterol load (85).

## VEGF-C

VEGF-C, the central factor of lymphangiogenesis, promotes lymphangiogenesis by binding with VEGFR-3 on the surface of LECs and regulates the physiological and pathological proliferation of lymphatic vessels. Compared to VEGFR-3, VEGF-C has less affinity with VEGFR-2, while binding with which can also promote angiogenesis. In addition to the effect on lymphatic and blood vessels, VEGF-C is closely associated with dyslipidemia and AS (86). A prospective study found that low levels of VEGF-C could independently predict all-cause mortality in patients with suspected or confirmed coronary artery disease (CAD) (87), while high levels of VEGF-C could degrade inflammation after MI, which was beneficial to the repair of injured myocardium (88).

### Source of VEGF-C

VEGF-C is highly expressed in embryonic tissues, such as syncytiotrophoblasts, cells in maternal decidua and endothelium of large placental vessels (89). In adults, VEGF-C is expressed in many parts including heart, lung, brain, kidney, intestine, and lymph nodes (90, 91).

### Mechanisms Regulating the Level of VEGF-C

#### Upregulation Mechanism of VEGF-C

Studies about the upregulation mechanism of VEGF-C in the cardiovascular field are relatively scarce. High salt (HS) diet could upregulate the TonEBP/VEGF-C signaling pathway, leading macrophage to infiltrate and accelerate the process of left ventricular remodeling in hypertension (92). Hyperglycemia could also induce the VEGF-C expression through LPA1/3, PLC, Akt, ROS, and LEDGF-dependent pathways (93).

Most relevant studies focus on tumors. For example, the oncoprotein PLCE1 of esophageal squamous cell carcinoma (ESCC) was observed to promote the growth and proliferation of tumor blood vessels by upregulating VEGF-C (94). Lysophosphatidic acid (LPA) also enhanced VEGF-C by stimulating LPA (1/3), COX-2, NF-κB, and EGFR trans activation-dependent mechanisms. Lymph markers were then increased, indicating these factors to be potential targets for controlling tumor metastasis (95). Higher VEGF-C stimulated by LPS promoted cell movement and lymphangiogenesis through TLR4-NF-κB/JNK signaling and then pushed the migration and invasion of colorectal cancer (CRC) (96). Noteworthily, oxLDL, as a risk factor of cardiovascular disease, was found to activate LOX-1-mediated NF-κB signaling pathway, lifted VEGF-C expression, and then promoted lymphatic metastasis of gastric cancer (97). In addition, there are related research in the field of cerebrovascular disease and skin disease. Theta pulse stimulation (CTBS) increased the expression of VEGF-C in cerebral meninges and promoted lymphangiogenesis (98). MiR-27b enhanced the expression of VEGF-C to promote the proliferation, migration, and angiogenesis of human microvascular endothelial cells (HMEC-1), thus conducing to angiogenesis and skin repair in scalded rats (99).

#### Downregulation Mechanism of VEGF-C

Because promoting lymphangiogenesis and angiogenesis can benefit the development of tumor, some studies have selected suppressing VEGF-C as a treatment to inhibit tumor growth, such as the highly specific RNA interference (RNAi) method (100). Through downregulating VEGF-C, miR-182-5p inhibited ERK and Akt signaling pathways to regulate angiogenesis and lymphangiogenesis, and partially regulate the tumorigenesis of colon cancer (101). Some drugs also present their direct suppressing effect on VEGF-C, like kolaviron in treating lymphatic filariasis (102) and norcantharidin in inhibiting lymphangiogenesis (103).

### Receptors and Target Cells

VEGFR-2 and VEGFR-3 are the receptors of VEGF-C, and by binding with VEGFR-3 can VEGF-C have a major biological effect. VEGFR-3 is expressed in lymphatic endothelium and high endothelial venules, which affects the differentiation, proliferation, migration, and survival of LECs. It is also expressed in other cells, such as osteoblasts, macrophages, and neural progenitor cells. VEGFR-3 helps to shape the lymphatic network during embryonic development and promote the formation of novel lymphatic vessels in adulthood. Inflammation or tumor may promote the expression of VEGF-C and VEGFR-3, and stimulate high endothelial venules to reform lymphatic vessels (104–106).

### Functions of VEGF-C

#### Inflammation and Lymphangiogenesis

The VEGF-C/VEGFR-3 signaling axis is of great importance to regulate the lymphatic system. Blocking the axis can interfere with lymphangiodilation and aggravate inflammation in a variety of disease models, including inflammatory bowel disease (IBD), rheumatoid arthritis, and skin inflammation. On the contrary, the targeted increase of VEGF-C can improve lymphangiodilation and reduce inflammation in such diseases (107). Many studies have proved that the cardiac lymphatic system can affect the regeneration potential of myocardium, making lymphangiogenesis a therapeutic target to accelerate the repair of injured heart (108, 109). After acute MI, VEGF-C stimulation conduce to the proliferation of cardiac lymphatic vessels, and transport immune cells to mediastinal lymph nodes (MLNs) through lymphatic vessel endothelial hyaluronic acid receptor-1 (LYVE-1), thus promoting the clearance of acute inflammatory response and cardiac repair (110). In addition, pre-treating lymphatic contractility with VEGF-C may prevent AS. As a study showed, early treatment with VEGF-C improved lymphatic molecule transport in LDLR –/– mice by lifting VEGFR-3 and FOXC2, and then limited plaque formation and macrophage accumulation (111). Some studies also illuminate that the inhibition or enhancement of VEGF-C can benefit hypertrophic cardiomyopathy. LCZ696, a new combination of angiotensin and enkephalinase inhibitor, could reduce the protein expression levels of VEGF-C, VEGFR3, and LYVE-1 in heart tissue of mice and improve the transport load of lymphatic vessels to macrophages, thus inhibiting the remodeling of lymphatic system in a hypertrophic cardiomyopathy model (112). However, a recent study also showed that the activation of the VEGF-C/VEGFR-3 axis prevented hypertrophic heart to heart failure (HF) and highlighted the selective stimulation of cardiac lymphangiogenesis as a potential treatment for hypertrophic cardiomyopathy (113). HS diet can also affect the level of VEGF-C. It was found that HS diet upregulated the TonEBP/VEGF-C signaling pathway, leading to severe macrophage infiltration and accelerating the process of left ventricular remodeling during hypertension (92). By contrast, overexpression of VEGF-C in HS mice promoted cardiac lymphangiogenesis, reduced myocardial fibrosis and macrophage infiltration, and preserved left ventricular function (114). In addition to the VEGF-C/VEGFR-3 pathway, the VEGFC/FLT4/PROX1 signal transduction axis could also promote the migration, proliferation, and differentiation of LECs, with hemopoietic expression homeobox (HHEX) as its upstream regulator (115).

#### Angiogenesis

VEGF-C regulates angiogenesis when binding with VEGFR-2. There is a link between angiogenesis and lymphangiogenesis; that is, angiogenesis will be significantly weakened when the lymphatic network is growing continually. Therefore, angiogenesis controlled by VEGF-C preferentially occurs far away from sites where lymphangiogenesis is happening. VEGF-C presents angiogenesis capacity through hydrolyzing and activating proteins in ECs. Intramyocardial injection of VEGF-C can induce the establishment of collateral circulation in ischemic myocardium (116). In addition, the angiogenic effect of VEGF-C is manifested in its influence on the development of coronary artery (CA). During the embryonic stage, VEGF-C plays a key role in the correct location and growth of CA stem on the aorta. In this process, VEGF-C is required to stimulate the vascular growth around the outflow tract and the absence of VEGF-C will lead to severe hypoplastic peripheral vessels in the heart, and delayed development and abnormal position of CA stem (117). Some specific forms of VEGF-C, like VEGF-C156S, lack angiogenesis function and only retain lymphangiogenesis function. In all, VEGF-C enjoys better lymphangiogenesis function than angiogenesis and improves lymphatic reflux disorder. Although it shows certain dilation effect on blood vessels, no evidence of angiogenesis or increased vascular permeability has ever been observed (118).

#### Anti-apoptosis

In the myocardial ischemia/reperfusion (I/R) injury model, myocardium pretreated with VEGF-C showed a lower MI area and a lower value of CK-MB, MDA, troponin, and apoptotic protein Bax. The mechanism lies in that VEGF-C activates Akt signaling pathway by binding to VEGFR-2 and blocks Bax expression and mitochondrial membrane translocation, thus inhibiting cardiomyocyte apoptosis and exerting cardioprotective effect after I/R injury (119).

#### Fibrogenesis

VEGF-C/VEGFR-3 significantly increased in a MI mice study. In addition to its regulatory effects on lymphatic vessels and inflammation, it directly promoted the proliferation, migration, and collagen synthesis of cardiac fibroblasts by activating TGF-β1 and ERK signaling pathways, and ultimately promoted myocardial fibrosis after MI (120).

### The Relation Between VEGF-C and Lipid Metabolism

The lymphatic system transports lipids from food that is absorbed by intestinal cells and packaged as CM into blood. The VEGF-C/VEGFR-3 pathway is involved in the lipid transport of intestinal lymphatic vessels, and dysfunction of the transport can lead to dyslipidemia. VEGF-C-deficient mice showed defected lipid absorption and increased excretion of fecal with dietary cholesterol and fatty acids. These mice also resisted obesity and improved glucose metabolism when fed with HFD. The mechanism may be related to intestinal lymphatic atrophy caused by VEGF-C deficiency (121). Another study observed that the inactivation of VEGFR-3 resulted in the retention of triglycerides (TGs) in the intestinal epithelial cells of the small intestine, reduced the postprandial level of plasma TGs, and increased the excretion of free fatty acids (FFA) and TGS into feces (122). Furthermore, systemic blockade of VEGFR-3 was demonstrated to reduce the infiltration of macrophages in adipose tissue and lipid accumulation in liver, and improve insulin sensitivity, revealing the unexpected effect of blocking VEGF-C and VEGF-D on the treatment of metabolic syndrome, which constituted a new therapeutic strategy for the prevention of obesity related insulin resistance (123). On the contrary, overexpression of VEGF-C transfer gene induced weight gain and insulin resistance in mice, and promoted the progress of metabolic syndrome (124).

Meanwhile, lipids can also influence the capacity of lymphatic vessels. Concretely, long-term HFD may cause adipose tissue expansion and the dysfunction of lymphatic system, especially collecting lymphatic vessels, further promoting the accumulation of adipose tissue (125).

## VEGF-D

VEGF-D, like VEGF-C, is a secretory glycoprotein. They share structural homology with each other and both promote lymphangiogenesis and angiogenesis by binding with VEGFR-2 and VEGFR-3. They emphasize on different functions, however, with VEGF-C being an essential factor for lymphangiogenesis, while VEGF-D compensates for the loss of VEGF-C during lymphangiogenesis and behaves more subtly. Besides, VEGF-D presents higher angiogenic potential than VEGF-C. A prospective study concluded that high levels of VEGF-D independently predicted all-cause mortality in patients with suspected or confirmed CAD (126). The increased VEGF-D was also associated with atrial fibrillation (AF) and ischemic stroke. The relationship between VEGF-D and ischemic stroke was more obvious in subjects diagnosed with AF (127).

### Source of VEGF-D

VEGF-D is highly expressed in the lung in the embryonic stage (128), while in adulthood, it is highly expressed in the heart, lung, and small intestine, but is expressed less in skeletal muscle, pancreas, and colon (129).

### Mechanisms Regulating the Level of VEGF-D

#### Upregulation Mechanism of VEGF-D

Inflammation can induce lymphangiogenesis. Studies have proved that inflammation can energize NF-κB to lift Prox1 and VEGFR-3. Then, the response of LECs to VEGF-C and VEGF-D is enhanced, which further promotes lymphangiogenesis (130). In a mice study, proinflammatory cytokine onterleukin-1β (IL-1β) activated NF-κB and then enhanced the production of VEGF-A, VEGF-C, and VEGF-D, ultimately inducing lymphangiogenesis in cornea (131). Higher VEGF-D levels can also result from a variety of tumor cells. For example, STAT3 in gastric cancer cells was found to promote lymphangiogenesis and lymph node metastasis by upregulating the expression of VEGF-D (132). Also, IL-7 could promote lymphatic proliferation and lymphatic spread in breast cancer cells by upregulating VEGF-D (133).

#### Downregulation Mechanism of VEGF-D

In terms of cardiovascular disease, the elevated level of VEGF-D in HF patients can be regulated to normal after heart transplantation (HT), which reflects the reversal of pulmonary congestion and the recovery of pulmonary artery compliance (PAC) and pulmonary vascular resistance (PVR) after HT (134). IL-1β is an increased proinflammatory cytokine in heart after MI. The factor is involved in downregulating VEGF-D in cardiac microvascular endothelial cells (CMECs) through ERK1/2, JNK, and PKCα/β, which then affects angiogenesis (135). Related studies about lung have shown that LPS induced lung injury suppressed the expression of VEGF-D, as well as VEGF-A, -B, -C, and VEGFR-1,−2,−3 (136). In addition, transforming growth factor-β1 (TGF-β1) abated VEGF-D expression in a dose- and time-dependent manner in human lung fibroblasts (137). In the field of cancer, sophocarpine was viewed to cut down the expression of VEGF-A, -C, and -D in CRC cells by decreasing N-cadherin, MMP-9, phosphorylated ERK, and phosphorylated MEK (138).

### Receptors and Target Cells

VEGF-D mediates angiogenesis and lymphangiogenesis by binding to VEGFR-2 and VEGFR-3 on ECs. VEGFR-2 is the core receptor of angiogenesis, and VEGFR-3 is essential for lymphangiogenesis. However, VEGFR-3 can also participate in angiogenesis by promoting the transformation of ECs from tip cell to stalk cell phenotype at the fusion point of vascular bud. Blocking VEGFR-3 can inhibit angiogenesis. Meanwhile, VEGFR-2 can also induce lymphatic vessel enlargement to regulate lymphangiogenesis. VEGF-D can also signal through the VEGFR-2/VEGFR-3 heterodimer, which can further boost the expansion of tumor collecting lymphatic vessels (139).

### Functions of VEGF-D

#### Lymphangiogenesis

Lymphangiogenesis is the cardinal biological effect of VEGF-D. In patients with aortic stenosis, the number of lymphatic vessels and the expression of VEGF-D, VEGFR-3, and VEGFR-2 in aortic valves were increased, indicating that lymphangiogenesis was induced in the progression of aortic stenosis (140). In a mice study, chronic overexpression of VEGF-D induced growth of novel lymphatic vessels in white adipose tissue and lymphangiodilation in brown adipose tissue, which benefited lipid metabolism and alleviated chronic inflammation. VEGF-D in white adipose tissue also increased macrophage infiltration and tissue fibrosis (141).

#### Angiogenesis

The mature form of human VEGF-D is an effective angiogenesis factor as it can increase the expression of VEGF-A, stanniocalcin-1 (STC1), and neuropilin (NRP)-2 to promote angiogenesis (142). Angiogenesis is the core of cardiac repair after MI. Cardiac angiotensin-converting enzyme (ACE)2 produces angiotensin (Ang)1–7, which in turn stimulates the expression of VEGF-D and MMP-9 in the heart to promote angiogenesis, thus conducing to heart recovery and improving ventricular function (143). As for treatment, the biological bypass of angiogenesis induced by VEGF-D gene therapy (GT) is a new concept for treating cardiac ischemia. Serotype 5 adenovirus is used to transfer VEGF-D cDNA to ischemic myocardium (144). A phase I/II a clinical trial showed that adenovirus VEGF-D ΔNΔC gene increased myocardial perfusion in the damaged area of myocardial perfusion reserve (145). Adenovirus-mediated VEGF-D gene transfer also presented excellent angiogenesis in rabbit skeletal muscle (146). In addition, VEGF-D-related angiogenesis is correlated to tumor progression. Cathepsin L (CTSL) could induce angiogenesis by regulating the CDP/Cux/VEGF-D pathway (147).

#### Fibrogenesis

VEGF-D can promote cardiac fibrosis, which regulates cardiac repair and remodeling. VEGF-D and VEGFR-3 were significantly upregulated in hearts of MI patients, especially in myofibroblasts at MI sites. A study proved that VEGF-D could stimulate the synthesis of MMP-2/-9 and tissue inhibitor of MMP (TIMP)-1/-2, and activate ERK phosphorylation, thus significantly increasing the proliferation and migration of cardiac fibroblasts. Last, the synthesis of type I collagen was significantly upregulated in a dose- and time-dependent manner, which promoted cardiac fibrosis (148).

#### Anti-apoptosis

VEGF-D may inhibit AS progression by its antioxidant virtue. Exogenous stimulation (such as hypochlorite) on ECs can be regulated by VEGF-D through mTOR kinase signal transduction to induce antioxidant response and maintain redox balance (149).

### The Relation Between VEGF-D and Lipid Metabolism

VEGF-D is an important regulator of lipid metabolism. Downregulation of VEGF-D/VEGFR3 signaling can decrease the genes that regulate the production of TGs and cholesterol, as well as the peroxisome β-oxidative pathway. In a LDLR –/– ApoB 100/100 mice study, knockout of VEGF-D gene could significantly increase plasma TGs and cholesterol and delay clearance of residual particles of large CMs that could not easily penetrate VECs. The mechanism was that inhibiting VEGF-D signal would reduce the expression of SCD1 (one of the main CMs residual receptors in LDLR deficiency) in the liver (150). In contrast, overexpression of VEGF-D in adipose tissue induced lymphangiogenesis in adipose tissue of mice, enhanced glucose clearance, and reduced insulin level and liver triglyceride, thereby resisting obesity-related immune accumulation and improving metabolic response (151).

## Conclusions

The VEGF family shows its extraordinary potential in the treatment of CHD by regulating angiogenesis, lymphangiogenesis, inflammation, apoptosis, redox, fibrogenesis, and lipid metabolism. Previous studies mostly focus on tumor and ophthalmic diseases, while studies about applying VEGF treating cardiovascular disease are relatively scarce. The following insights into the VEGF-related direction of treating CHD may be illuminating in the obscure field. The VEGF family interferes with humans in a complicated way because these factors present duality in their functions and the outcomes in target tissues can also be opposite with the stimulation of an identical factor. Concretely, a certain degree of angiogenesis conduces to cardiac recovery from hypoxia, while an excessive amount of it may push AS plaques to an unstable state. Different isoforms of VEGF-A can vary in quantities in disparate environments and thus display an overall angiogenetic or anti-angiogenetic function. Researchers can analyze the dynamics of the isoforms in CHD patients and locate the favorable realm by incorporating their health condition. Then, the realm may be set for a treatment target for CHD and a predictor for prognosis. Regarding lymphangiogenesis, the VEGF family is closely tied to the lymphatic system, which further regulates inflammation, redox, lipid metabolism, and so on, while clinical studies rarely extract lymph from patients to test its component, let alone the different composition between CHD and healthy people. Their level in lymph may not be comparable to that in serum. In addition to lymph, the lymphatic system in the intestinal tract makes another attraction due to its intimate association with lipid metabolism. The imaging of the intestinal lymphatic system may be a possible way for predicting VEGF level, lipid level, and CHD progression. Therefore, by summarizing and assorting functions and mechanisms of the VEGF family, we hope that this study can provide help for researchers who start from the VEGF family to find therapeutic targets for CHD.

## Author Contributions

YZ chose the title, assorted information, and drafted the manuscript. XZ drafted the manuscript. HC offered advice about the structure. JS, GY, and SS assorted information. YH governed the whole process and offered advice. All authors contributed to the article and approved the submitted version.

## Conflict of Interest

The authors declare that the research was conducted in the absence of any commercial or financial relationships that could be construed as a potential conflict of interest.

## Publisher's Note

All claims expressed in this article are solely those of the authors and do not necessarily represent those of their affiliated organizations, or those of the publisher, the editors and the reviewers. Any product that may be evaluated in this article, or claim that may be made by its manufacturer, is not guaranteed or endorsed by the publisher.
